# Targeting CD38 in acute myeloid leukemia interferes with leukemia trafficking and induces phagocytosis

**DOI:** 10.1038/s41598-021-01300-8

**Published:** 2021-11-11

**Authors:** Meike Farber, Yiyang Chen, Lucas Arnold, Michael Möllmann, Eva Boog-Whiteside, Yu-An Lin, H. Christian Reinhardt, Ulrich Dührsen, Maher Hanoun

**Affiliations:** 1 Department of Hematology and Stem Cell Transplantation, University Hospital Essen, Hufelandstraße 55, 45122 Essen, Germany; 2 Division of Hematology and Oncology, Department of Medicine, Chang Gung Memorial Hospital, Chiayi, Taiwan

**Keywords:** Cancer microenvironment, Oncology, Acute myeloid leukaemia

## Abstract

Targeting the interaction between leukemic cells and the microenvironment is an appealing approach to enhance the therapeutic efficacy in acute myeloid leukemia (AML). AML infiltration induces a significant release of inflammatory cytokines in the human bone marrow niche which accelerates leukemogenesis. As the transmembrane glycoprotein CD38 has been shown to regulate cytokine release, we assessed the anti-leukemic potential of CD38 inhibition in AML. CD38 expression in AML cells proved to depend on microenvironmental cues and could be significantly enforced through addition of tretinoin. In fact, the anti-CD38 antibody daratumumab showed significant cytostatic efficacy in a 3D in vitro triple-culture model of AML, but with modest cell-autonomous cytotoxic activity and independent of CD38 expression level. In line with a predominantly microenvironment-mediated activity of daratumumab in AML, CD38 inhibition significantly induced antibody-dependent phagocytosis and showed interference with AML cell trafficking in vivo in a xenograft transplantation model, but overall lacked robust anti-leukemic effects.

## Introduction

Acute myeloid leukemia (AML) is the most common acute leukemia in adults with increasing incidence with rising age^1^. Already at low disease burden, AML is marked by severe hematopoietic failure, which is the major cause of death. Additionally, despite intensive chemotherapeutic protocols, high relapse rates and primary resistance frequently occur, requiring further treatment intensification, including allogeneic hematopoietic stem cell transplantation^1^. However, many elderly AML patients are not eligible for intensive treatment protocols and therefore show disappointingly low overall survival rates with few patients surviving beyond five years of follow-up^2^. A major obstacle in AML therapy is persistence of quiescent leukemia-initiating cells, which are protected in their bone marrow niche^3^. Therefore, uncovering the microenvironmental regulation of AML cells and defining, ideally less toxic, niche-targeted therapies is of utmost need to make leukemia cells more vulnerable to genotoxic drugs and efficiently eradicate residual leukemia cells. Recently, we have gained increasing insight how AML cells are regulated in the bone marrow niche in murine models and humans^4–6^. AML infiltration induced a significant release of inflammatory cytokines in the human bone marrow, which proved to accelerate leukemogenesis^7^. The transmembrane glycoprotein CD38 appears to regulate cytokine release, adhesion, and cellular migration toward sites of inflammation^8^. CD38 is widely expressed on immune cells and its expression level depends on the maturation and activation state^9^. Besides, most AML blasts show high CD38 expression without obvious correlation with cytomorphological and genetic characteristics^10^. Indeed, CD38 monoclonal antibodies have shown high efficacies in multiple myeloma at different lines of therapy, which is exerted through both antibody-mediated cytotoxicity, as well as modulation of immune surveillance in the niche^11,12^. Notably, the first-in-class human IgG1κ anti-CD38 antibody daratumumab showed, throughout a number of clinical studies, a beneficial spectrum of side effects. Therefore, inhibition of CD38 in AML appears to be an appealing concept. Here, we show that CD38 inhibition leads to significant cytostatic efficacy in a 3D in vitro triple-culture model of AML, with modest cell autonomous cytotoxic activity. Daratumumab significantly induces antibody dependent phagocytosis in AML and shows interference with AML cell trafficking in vivo in a xenograft transplantation model.

## Results

### CD38 inhibition results in high anti-leukemic efficacy in vitro independent of CD38 expression level

**Figure 1:**
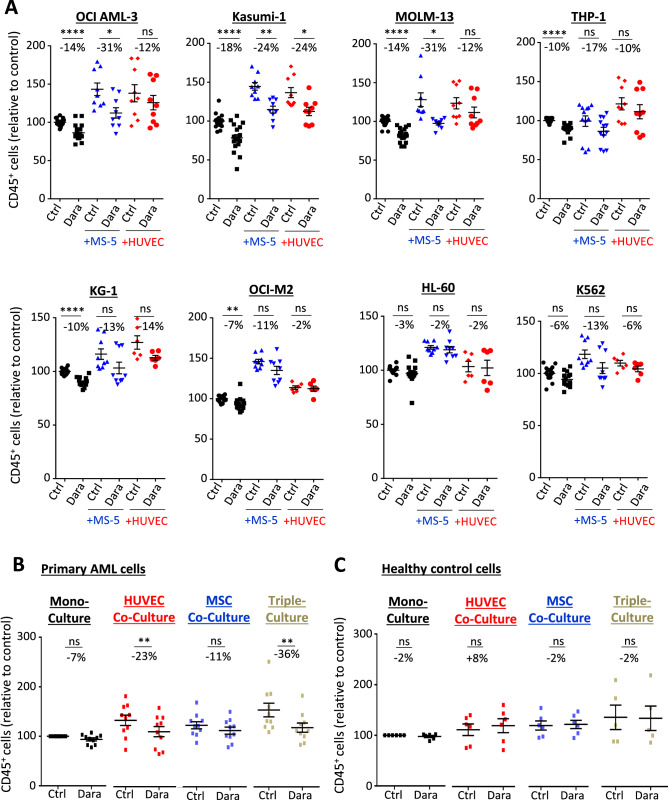
CD38 inhibition results in high anti-leukemic efficacy in vitro independent of CD38 expression level. (**A**) Human AML cell lines were either mono- or co-cultured with MS-5 stroma cells or HUVEC cells and treated with 0.1 µg/ml daratumumab or vehicle for 4 days. Absolute numbers of CD45^+^ cells were normalized to mono-cultured control, for each pair the mean change in cell count is given in percentage (n = 3). (**B)** Primary AML cells (n = 10) or **(C)** healthy peripheral blood mononuclear cells (n = 6) were mono-, co- or triple-cultured with HUVEC and/or mesenspheres and treated with 0.1 µg/ml daratumumab or vehicle for 3 days. Absolute numbers of CD45^+^ cells were normalized to mono-cultured control. Each dot represents the mean of triplicates, for each pair the mean change in cell count is given in percentage. Data are shown as mean ± SEM. n.s., not significant, *p < 0.05 **p < 0.01 ***p < 0.001 ****p < 0.0001 as determined by unpaired student´s t-test (**A**) and Wilcoxon signed-rank test (**B**,**C**). See also Supplementary Fig. S1.

To test if CD38 inhibition affects leukemia growth in AML, we applied the monoclonal antibody daratumumab on a series of different AML cell lines in mono-culture, as well as in co-culture to elaborate putative niche-mediated effects of daratumumab. AML cell lines were cultured with endothelial cells (human umbilical vein endothelial cells, HUVEC) or MS-5 fibroblasts, which have been shown before to significantly support AML cell line growth^7^. We could confirm that co-culture with stroma cells led to an increased expansion of AML cells (Fig. 1a). Daratumumab showed robust, but only minor cytotoxic effects on mono-cultured AML cells, which could not be significantly enhanced with increasing dosages of up to 10 µg/ml (Fig. 1a, Supplementary Fig. S1a). In contrast, in co-cultured AML cells daratumumab displayed increased anti-leukemic activity already at 0.1 µg/ml, in particular for OCI-AML3, Kasumi-1 and MOLM-13 cells. Only minor anti-leukemic effects were observed in KG-1, OCI-M2, HL-60 and K562 cells. To test its clinical relevance, we treated a number of primary human AML or peripheral blood mononuclear cells from healthy donors with 0.1 µg/ml daratumumab. To better mimic the influence of the microenvironment on primary human AML cells, we co-cultured hematopoietic cells with either HUVEC or mesenchymal stem and progenitor cells grown as non-adherent spheres, hereafter referred to as mesenspheres, or both under serum-free conditions. Indeed, we observed that co-culture with either stroma cell type promoted growth of primary hematopoietic cells indicating that our in vitro system partially recapitulates features of the hematopoietic stem cell niche (Fig. 1b,c). Applying daratumumab on AML cells co-cultured with endothelial cells or mesenspheres resulted in significant reduction in AML cell numbers (Fig. 1b). The anti-leukemic effect was even more pronounced when culturing AML cells in 3D triple-culture with adherent endothelial cells and mesenspheres, where daratumumab induced reduction in AML cell number by 36%. Again, no anti-leukemic effects were observed in mono-cultured AML cells. Of note, the anti-leukemic efficacy did not vary between AML samples of different genetic risk characteristics categorized according to the European LeukemiaNet recommendations^1^ (Supplementary Table 1). Further, we did not observe any substantial cytotoxic effects of daratumumab on healthy peripheral blood mononuclear cells (Fig. 1c) nor on stroma cells (Supplementary Fig. S1b). To exclude any unspecific effects of daratumumab, we applied next to media containing the carrier solution, IgG_1_ as well as IgG_1_-b12 monoclonal antibody as isotype controls and did not observe any cytotoxic effects in any control condition compared to daratumumab (Supplementary Fig. S1c). Further, similar cytostatic effects were observed when AML cells were treated with isatuximab, another anti-CD38 antibody (Supplementary Fig. S1d,e).

**Figure 2:**
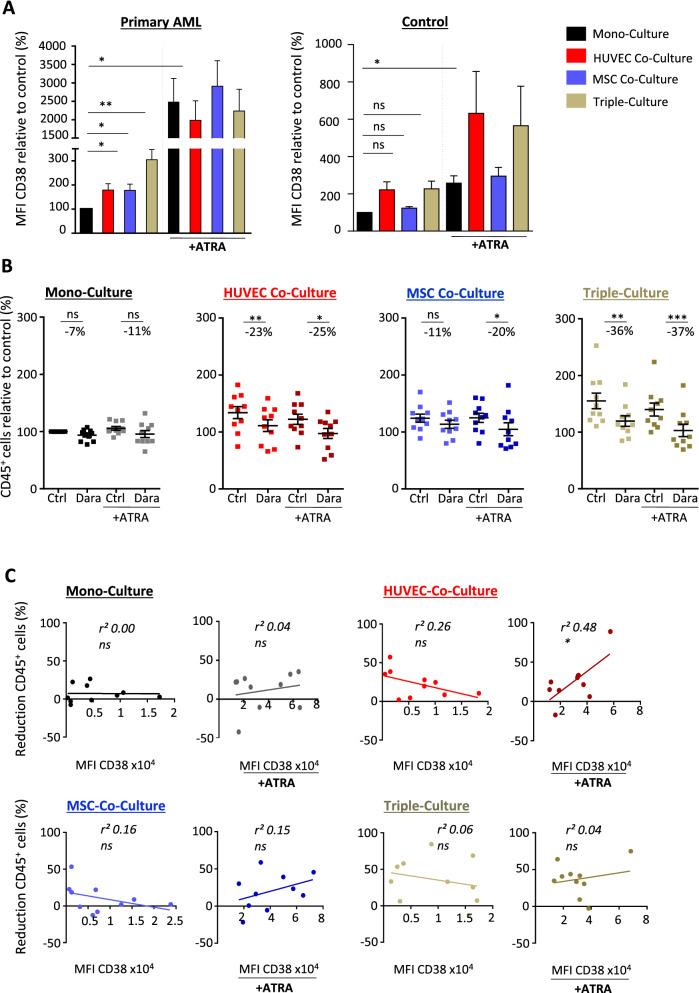
CD38 expression of AML cells is regulated by microenvironmental cues and induced upon ATRA treatment. (**A**) Mean fluorescence intensity (MFI) of CD38 on primary AML cells (n = 10) and healthy peripheral blood mononuclear cells (n = 6) measured by flow cytometry, mono-, co- or triple-cultured with HUVEC and/or mesenspheres before and after treatment with 0.1 µM all-trans-retinoic acid (ATRA), data normalized to mono-cultured control. (**B**) primary AML cells (n = 10) were mono-, co- or triple-cultured with HUVEC and/or mesenspheres. ATRA or vehicle was added at 0.1 µM for 2 days followed by daratumumab treatment at 0.1 µg/ml or vehicle for another 3 days. Absolute numbers of CD45^+^ cells were normalized to mono-cultured control. Each dot represents the mean of triplicates, for each pair the mean change in cell count is given in percentage. (**C**) Correlation of anti-leukemic activity of daratumumab given as mean cell-count reduction with CD38 expression given as MFI measured by flow cytometry for each primary AML sample, mono-, co- or triple-cultured with HUVEC and/or mesenspheres (n = 10 different AML donors, each primary sample was tested in triplicate). Data are shown as mean ± SEM. n.s., not significant, *p < 0.05 **p < 0.01 ***p < 0.001 as determined by paired one-way ANOVA (A), Wilcoxon signed-rank test (B) and linear regression (C). See also Supplementary Fig. S2.

Given the fact that daratumumab harbored AML-specific anti-leukemic activity, which was predominantly induced through microenvironment-mediated effects, we next asked whether CD38 expression on AML cells is influenced by microenvironmental cues and if the anti-leukemic activity of daratumumab depends on CD38 expression levels. Co-culture of AML cells with both HUVEC and mesenspheres led to a statistically significant three-fold increase of CD38 expression in AML cells, but no significant difference in healthy peripheral blood mononuclear cells (Fig. 2a). It has been shown that the CD38 gene contains a retinoic acid responsive element, which, upon treatment with ATRA, induces CD38 upregulation^13^. In fact, treatment with 0.1 µM ATRA led to a 25-fold higher CD38 expression in AML cells independent of the culture condition, while healthy control cells only showed a three to sixfold increased expression (Fig. 2a). Adding ATRA to mono-, co- or triple-cultured primary human AML cells or mononuclear control cells did not lead to significantly increased anti-leukemic effects of daratumumab (Fig. 2b, Supplementary Fig. S2a). To further elaborate the dependency of the anti-leukemic activity of daratumumab on CD38 expression in AML, we correlated the CD38 expression of primary AML cells under different culture conditions with the degree of reduction of AML cell growth in vitro (Fig. 2c). We did not observe any consistent correlation of CD38 expression and anti-leukemic efficacy of daratumumab; only in primary AML cells co-cultured with HUVECs in the presence of ATRA the anti-leukemic activity of daratumumab depended on CD38 expression. Similarly, we did not observe robust correlation between CD38 expression and sensitivity to daratumumab in AML cell lines, mono- or co-cultured with HUVECs (Supplementary Fig. S2b). However, co-culture with MS-5 showed a significant correlation of CD38 expression and sensitivity to daratumumab treatment.

In summary, daratumumab showed robust, AML-specific anti-leukemic efficacy in vitro, which is at least partially mediated through microenvironmental cues and to a lower extent through direct cytotoxic effects. CD38 expression in AML cells is regulated through the microenvironment and can be significantly enforced through addition of ATRA, while the anti-leukemic efficacy of daratumumab does not stringently depend on the level of CD38 expression.

### Daratumumab induces phagocytosis in AML

**Figure 3:**
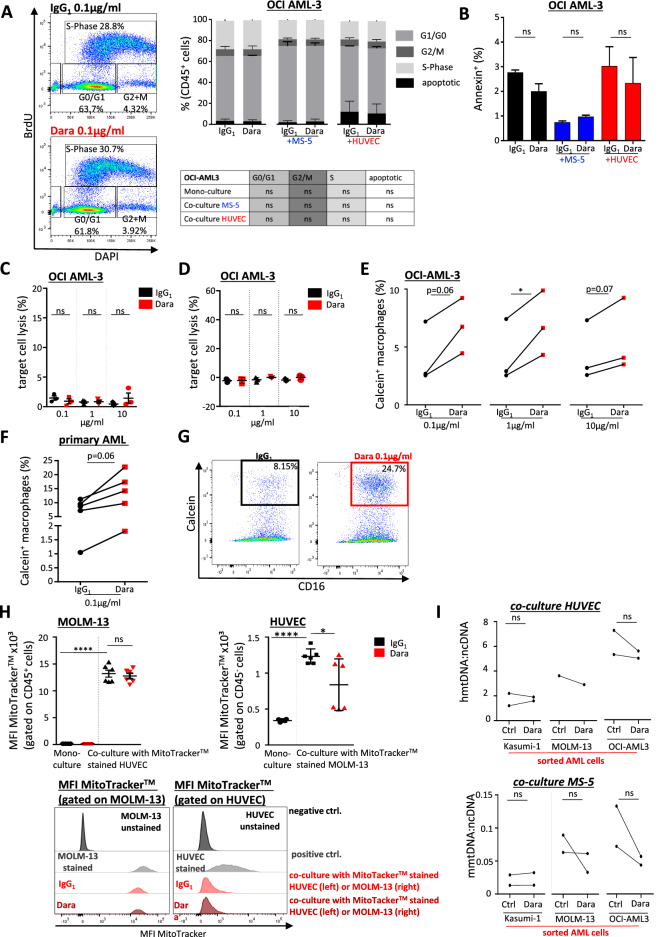
Daratumumab induces phagocytosis in AML. (**A**) Cell proliferation analysis using BD BrdU flow assay. Left, representative flow cytometry plots, right quantification for OCI-AML-3 cells mono- or co-cultured with MS-5 or HUVEC after treatment with 0.1 µg/ml daratumumab or IgG_1_ control for 4 days, statistical analyses are given below (n = 3 independent experiments). (**B**) Quantification of apoptotic cells by Annexin V assay of OCI-AML-3 cells mono- or co-cultured with MS-5 or HUVEC after treatment with 0.1 µg/ml daratumumab or IgG_1_ control for 4 days (n = 3 independent experiments). (**C**) Quantification of complement-dependent-cytotoxicity (CDC). Calcein stained OCI-AML-3 cells were incubated in medium supplemented with 10% normal human serum and treated with increasing dosages of daratumumab or IgG_1_ control for 1 h. (**D**) Quantification of antibody-dependent cell-mediated cytotoxicity (ADCC). Calcein stained OCI-AML-3 cells were incubated with effector cells and treated with increasing dosages of daratumumab or IgG_1_ control. For CDC and ADCC, calcein-fluorescence was measured in supernatant by spectrophotometry. Lysis was calculated by using detergent as maximum and medium as minimum control (n = 3 independent experiments). (**E–G**) Quantification of antibody-dependent phagocytosis (ADCP). Macrophages were cultured with calcein stained OCI-AML-3 (n = 3 independent experiments) (**E**) as well as primary AML cells (n = 5 different AML donors, each primary sample was tested in triplicate) (**F**) and treated with increasing dosages of daratumumab or IgG_1_ control. CD16^+^ calcein^+^ phagocyting macrophages were measured by flow-cytometry. Each dot represents the mean of triplicates. (**G**) Representative flow cytometry plot of one calcein stained primary AML sample co-cultured with macrophages showing the fraction of AML-phagocyting macrophages. (**H**) Tracking mitochondrial transfer in co-culture was performed by staining mitochondria with MitoTracker™. Left**,** MFI of MitoTracker™ in MOLM-13 cells after co-culture with MitoTracker™ stained HUVEC and treatment with 0.1 µg/ml daratumumab or IgG_1_ control for 24 h or mono-culture of unstained MOLM-13 as control. Right**,** MFI of MitoTracker™ in HUVEC after co-culture with MitoTracker™ stained MOLM-13 cells and treating with 0.1 µg/ml daratumumab or IgG_1_ control for 24 h or mono-culture of unstained HUVEC as control. Below**,** representative histogram of MFI of MitoTracker™, gated on MOLM-13 cells (left) or HUVEC (right) at the indicated culture conditions and treatment with 0.1 µg/ml daratumumab or IgG_1_ control. (**I**) Indicated AML cells were co-cultured with either HUVEC (top) or MS-5 cells (bottom) for 24 h, sorted by flow cytometry and mRNA expression levels were measured by rt-PCR for either human mitochondrial DNA *(hmtDNA)* after HUVEC co-culture or mouse mitochondrial DNA *(mmtDNA)* after MS-5 co-culture, relative to ncDNA. Data are shown as mean ± SEM. n.s., not significant, *p < 0.05****p < 0.0001 as determined by unpaired student´s t-test (**A**, **B**, **E**, **H**, Mann–Whitney-U Test (**C**, **D**, **I**), and Wilcoxon signed-rank test (**F**). See also Supplementary Fig. S3-5.

To further elucidate the underlying mechanisms of the anti-leukemic activity of daratumumab in AML, we assessed the effects of daratumumab on proliferation in the most drug-responsive AML cell lines. BrdU analysis did not show significant differences in proliferation upon treatment (Fig. 3a, Supplementary Fig. S3a). Neither did we observe an induction of apoptosis by annexin quantification by flow cytometry (Fig. 3b, Supplementary Fig. S3b) nor by measuring mRNA levels of apoptosis-related genes (Supplementary Fig. S3c). Furthermore, daratumumab did not induce differentiation of AML cells as assessed by cytomorphology (data not shown). As daratumumab has been shown to induce multiple immune-mediated cytotoxic effects in multiple myeloma^12^, we next aimed at assessing these mechanisms in AML. We evaluated daratumumab-induced complement-dependent cytotoxicity (CDC) in OCI-AML3 and Kasumi-1 cells as the most drug-responsive AML cell lines, which were preincubated with calcein-AM and treated with increasing concentrations of daratumumab or isotype control. Here, daratumumab did not induce significant cell death in the presence of normal human serum (10%) (Fig. 3c and Supplementary Fig. S3d). Nor could we detect any capacity of daratumumab to induce antibody-dependent cellular cytotoxicity (ADCC) by FcγR-bearing effector cells at different drug concentrations in AML (Fig. 3d and Supplementary Fig. S3e). To evaluate the capacity to induce antibody-dependent cellular phagocytosis (ADCP) in AML, we generated macrophages from peripheral blood-derived monocytes stimulated with M-CSF and co-cultured with calcein-AM labeled AML cells in the presence of daratumumab or isotype control at the indicated concentrations. Daratumumab induced ADCP with increased phagocytosis of OCI-AML3 of around 1.64-fold (Fig. 3e) and for Kasumi-1 cells of 1.7-fold (Supplementary Fig. S3f.), which was not dose-dependent. To further substantiate these findings, we also applied primary human AML cells and documented for all five samples an increased phagocytosis of around 1.74-fold after treatment with daratumumab (Fig. 3f-g). In summary, in accordance to the minor cell-autonomous anti-leukemic effects of daratumumab in AML, we did not observe any significant impact on AML proliferation, apoptosis, differentiation nor CDC and ADCC, while daratumumab strikingly induced phagocytosis of AML cells. As endothelial cells are known to harbor phagocytic capacities^14^, this might explain the significant microenvironment-mediated anti-leukemic effects of daratumumab in our 3D triple-culture model. Next, we evaluated whether daratumumab interferes in the interaction of mesenchymal stem and progenitor cells with AML cells. It has been previously shown that AML cells communicate with stroma cells through transfer of mitochondria^15^. In multiple myeloma mitochondrial transfer occurs through formation of nanotubules which was dependent on CD38 expression^16^. To test whether daratumumab disrupts the mitochondrial transfer and herewith the communication of AML cells with stroma cells, we traced mitochondria using MitoTracker™ dye. In fact, we could prove that stroma cells transfer significant amounts of mitochondria to AML cells, while stroma cells only incorporate a limited amount of AML-derived mitochondria (Fig. 3h, Supplementary Fig. S4a-b). Adding daratumumab to MOLM-13, OCI-AML-3 or Kasumi-1 cells co-cultured with HUVECs or MS-5 did not affect mitochondria transfer between AML and stroma cells (Fig. 3 h, Supplementary Fig. S4a-b). Due to possible dye efflux, we also quantified mtDNA in sorted AML cells after co-culture with HUVEC or MS-5 cells. After one day of co-culture there was a trend for decreased mitochondrial transfer upon daratumumab treatment, which disappeared after three days (Fig. 3i, Supplementary Fig. S4c).

As human CD38 was shown to bind to CD31, which is highly expressed on endothelial cells, we assumed a putative role in interference with adhesion and circulation of leukemia cells^17^. Therefore, we analyzed the gene expression profile of endothelial and hematopoietic cells purified by flow cytometry after triple-culture with either AML cells or control mononuclear cells. We assessed the expression of genes associated with migration and retention (*CXCR4, CXCL12*), adhesion (*ICAM-1, VCAM-1, E-selectin, PECAM-1*) and angiogenesis-related factors (*Angiopoietin-1, VEGF, VE-cadherin*) in mono- or co-cultured cells treated with either ATRA, daratumumab or both. However, we did not observe any differences in mRNA levels neither in AML cells nor endothelial cells co-cultured with either AML or control peripheral blood mononuclear cells after treatment with daratumumab (Supplementary S5a-b). In conclusion, the microenvironment-mediated anti-leukemic activity of daratumumab is not due to interference with mitochondrial transfer nor in the expression of adhesion-, migration- and angiogenesis-related factors in vitro.

### Daratumumab interferes with AML cell trafficking

**Figure 4:**
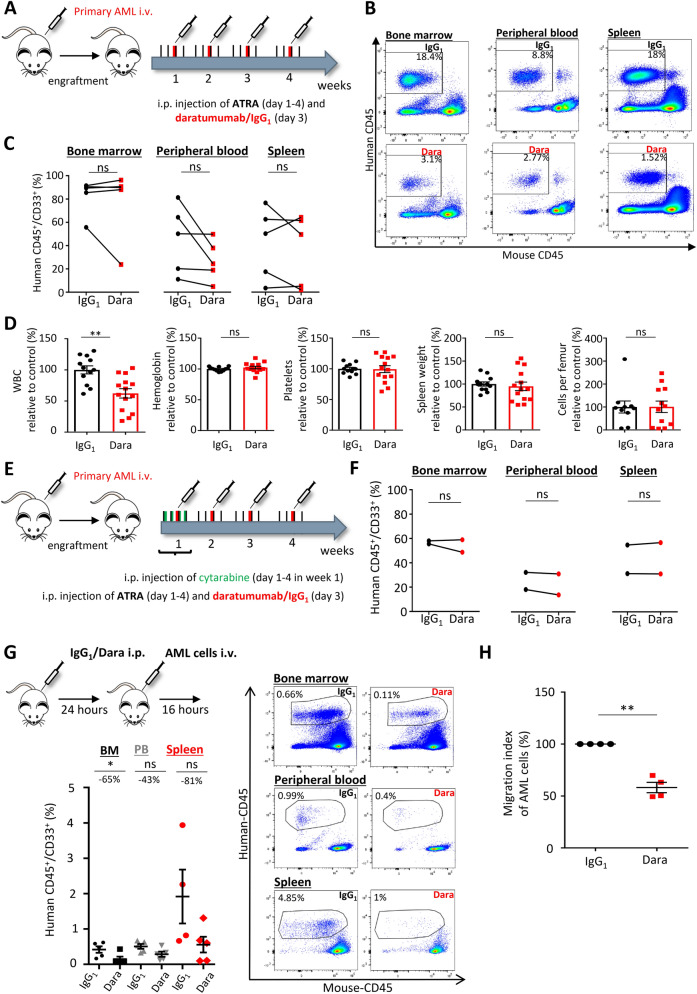
Daratumumab interferes in AML cell trafficking*.* (**A**) Schematic illustration of in vivo treatment protocol with ATRA (10 mg/kg) for all mice and daratumumab/IgG_1_ (8 mg/kg) after engraftment of primary human AML cells in NSG mice. (**B**) Representative flow cytometry plots of human hematopoietic engraftment by gating on human CD45^+^ cells in bone marrow, peripheral blood and spleen of mice treated with daratumumab or IgG_1_ control. (**C**) Quantification of human AML engraftment (CD45^+^/CD33^+^ cells) in peripheral blood, bone marrow and spleen. Every dot represents mean of all mice per AML sample, n = 5 different primary AML samples and n = 1-5 mice per sample per group. (**D**) White blood cells count, hemoglobin levels, platelet count, wet spleen weight and cells per femur of daratumumab or IgG_1_ treated mice (data normalized to control, n = 26). (**E**) Schematic illustration of treatment protocol with cytarabine (20 mg/kg), ATRA (10 mg/kg) and daratumumab/IgG_1_ (8 mg/kg) after engraftment of primary human AML cells in NSG mice. (**F**) Quantification of human AML engraftment (CD45^+^/CD33^+^ cells) in peripheral blood, bone marrow and spleen. Every dot represents mean of all mice per AML sample, n = 2 different AML samples, 2–3 mice per sample per group. (**H**) Top, experimental layout to test for interference in AML homing. Below, human AML engraftment (CD45^+^/CD33^+^ cells) in bone marrow (BM), peripheral blood (PB) and spleen 16 h after transplantation, n = 5 mice per group, 2 different AML samples. Right, representative flow cytometry plots of human hematopoietic engraftment by gating on human CD45^+^ cells in bone marrow, peripheral blood and spleen for each experimental condition. (**H**) Quantification of transendothelial migration of primary AML cells (n = 4, mean of duplicates). Shown is migration index calculated by cells migrated/total cells seeded, normalized to IgG_1_ control (each dot represents the mean of triplicates). Data are shown as mean ± SEM. n.s., not significant, *p < 0.05, **p < 0.01 as determined by Wilcoxon signed-rank test (C), paired students t-test (D, G) and Mann–Whitney-U Test (F, H). See also Supplementary Fig. S6.

To test the anti-leukemic activity of daratumumab in a clinically relevant in vivo model, we transplanted different primary human AML cells into immunodeficient NSG mice. As shown before, secondary transplantation of primary human AML cells allowed robust engraftment without any conditioning and, therefore, avoiding additional influence on the microenvironment^7^. Given the robust upregulation of CD38 expression in AML upon ATRA treatment (Fig. 2a), all mice were treated with ATRA in addition to daratumumab or IgG_1_ control (Fig. 4a). With a mean leukemia burden of ~ 7% (mean ± 1) in the peripheral blood, mice were randomized and weekly treated on 4 consecutive days with ATRA (10 mg/kg), as well as daratumumab (8 mg/kg) or IgG_1_ isotype control on the third day of each week for in total 4 weeks and analyzed 6 days after the end of treatment (Fig. 4a). Indeed, we could prove that addition of ATRA induced CD38 expression in AML cells in IgG_1_-treated control mice*,* although to a lower extend than in vitro (Supplementary Fig. S6a). In 3 out of 5 experiments we observed a striking decrease in human AML cells in peripheral blood by -30%, while overall no significant reduction in leukemia burden was observed in bone marrow and spleen (Fig. 4b,c, Supplementary Fig. S6b). In line with the apparent anti-leukemic activity observed in the peripheral blood, leukocyte counts were significantly lower in daratumumab-treated mice (Fig. 4d). After daratumumab treatment, bone marrow cellularity was slightly increased, while we did not observe obvious histological differences in bone marrow architecture, in particular no obvious differences in the amount of reticular fibers (Fig. 4d, Supplementary Fig. S6c). Notably, within one experiment, we observed a stronger anti-leukemic activity of daratumumab, where the average leukemia burden in the control group was with 55% AML infiltration comparably low. With the assumption that the anti-leukemic activity of daratumumab might be more pronounced at lower leukemia burden, we combined daratumumab treatment with a chemotherapeutic agent. For that, we added 20 mg/kg cytarabine on 4 consecutive days of the first week of treatment to the above-mentioned scheme (Fig. 4e). While the overall leukemia burden was comparably low, daratumumab treatment did not result in any significant differences in leukemia burden nor cellularity compared to IgG_1_ isotype treated controls (Fig. 4f, Supplementary Fig. S6d). Given the obvious redistrubition of leukemia cells after daratumumab treatment, we wondered if CD38 inhibition affects AML cell trafficking in vivo. For that, we treated NSG mice with daratumumab (8 mg/kg) or IgG_1_ isotype control and 24 h later mice were transplanted with primary human AML cells. Sixteen hours after transplantation, we found three- to five-fold less AML cells in bone marrow and spleen, respectively, indicating an impaired homing of AML cells after daratumumab treatment (Fig. 4g). To elucidate the effects of daratumumab on AML/endothelial cell interactions, we assessed the transendothelial migration capacity of primary human AML cells. In fact, daratumumab significantly impaired transendothelial migration of AML cells by almost 50%, while a direct cytotoxic effect after 16 h was ruled out (Fig. 4h, Supplementary Fig. S6e). Nevertheless, we cannot completely rule out that the lower number of AML cells in bone marrow and spleen after transplantation are due to immune-mediated cytotoxic effects of daratumumab. In summary, CD38 inhibition interferes in AML cell trafficking resulting in lower leukemia burden particularly in the peripheral blood, but overall does not show robust anti-leukemic activity as monotherapy or in combination with cytarabine in vivo.

## Discussion

Disrupting the microenvironmental interplay with AML cells is an appealing approach to target leukemia-initiating cells and putatively increases their sensitivity to genotoxic influences. Both cell-autonomous and microenvironment-derived inflammatory signals promote clonal hematopoiesis within an aged bone marrow microenvironment and facilitate leukemogenesis, as for instance in B lineage malignancies as well as myeloid neoplasms^18,19^. Targeting inflammatory signals mitigates clonal hematopoiesis in *TET2*-mutated preleukemic stem and progenitor cells^20^, blocking IL-6 signaling delays chronic myeloid leukemia development^21^ and interfering in the JAK/STAT pathway shows anti-leukemic activity in AML in vitro^7^, which prove the feasibility of targeting the inflammasome in myeloid diseases. Based on the prominent immunomodulatory function of CD38 and its wide expression in AML, we elaborated the effects of CD38 inhibition in AML. Daratumumab showed significant anti-leukemic activity in a 3D triple culture model, with only minor cell-autonomous efficacy, suggesting that daratumumab´s anti-leukemic effects are predominantly mediated through the microenvironment. We could prove that CD38 expression in AML cells is regulated by niche cells and significantly increases after ATRA treatment. However, in contrast to a previous study, combination of daratumumab and ATRA did not increase anti-leukemic effects^22^. In line with the obvious microenvironment-mediated anti-leukemic activity, we found that daratumumab elicits phagocytosis of AML blasts and seems to interfere with AML circulation. CD38 was shown before to regulate migration of chronic lymphocytic leukemia cells through interaction with its ligand CD31^23^, which is highly expressed on endothelial cells and which are significantly increased in AML bone marrow^24^. In this regard, it remains unclear how daratumumab interferes with the communication of AML cells with mesenchymal stroma cells, at least in our model, transfer of mitochondria is not significantly affected. Applying daratumumab in vivo in a xenograft transplantation model with primary human AML cells did not lead to robust anti-leukemic effects. In contrast, daratumumab showed significant anti-leukemic efficacy in a subcutaneous scaffold implanted with primary human AML cells^25^ and xenograft transplantation model with AML cell lines with early therapeutic intervention^26^. Based on the hypothesis that daratumumab might exert its highest anti-leukemic efficacy at low disease burden, we combined daratumumab with a chemotherapeutic agent, but were still missing robust anti-leukemic effects. Given the diversified immunomodulatory function of CD38, it remains unclear to what extent the actual efficacies of daratumumab in AML are recapitulated in immunodeficient NSG mice lacking functional B cells, T cells and natural killer cells. Furthermore, lacking human phagocytizing cells in NSG mice could explain the missing anti-leukemic activity of daratumumab. Therefore, further investigation to find the appropriate model and context to test daratumumab in AML is still required.

## Methods

### Patient samples

Primary patient samples were obtained from peripheral blood of newly diagnosed AML patients and non-leukemic donors after informed consent of all subjects according to institutional guidelines. The study was carried out in accordance with relevant guidelines and regulations, with an approved protocol of the University of Duisburg-Essen ethics committee. Mononuclear cells (MNC) were purified using Lymphoprep™ (STEMCELL Technologies, Vancouver, Canada). Patient characteristics are summarized in Supplemental Table 1.

### In vivo treatments

All animal experiments were approved by the responsible local government authorities, namely the Landesamt für Natur, Umwelt und Verbraucherschutz Nordrhein Westfalen. All animal experiments were carried out in accordance with the relevant guidelines and regulations and complied with the ARRIVE guidelines. NOD-scid Il2Rg^−/−^ (NSG) mice were bred and used in the animal care facility at the University Hospital Essen. Human mononuclear cells derived from untreated AML patients were depleted from CD3^+^ cells using 2 µl OKT3/1 × 10^6^ cells (BioLegend, San Diego, USA) and intravenously transplanted into NSG mice. Bone marrow and spleen cells were harvested from leukemic mice and used for secondary transplantation without prior conditioning. Leukemic mice were equally randomized according to their peripheral blood leukemia burden to each treatment group. Daratumumab (8 mg/kg) or IgG_1_ control antibody were given once weekly intraperitoneally for 4 weeks, while all-trans-retinoic acid (ATRA) was administered at 10 mg/kg intraperitoneally for 4 consecutive days per week for a total of 4 weeks (Fig. 4a). In a second experimental approach, cytarabine was added to the first week of treatment, administering 20 mg/kg intraperitoneally for 4 consecutive days (Fig. 4e). Six days after end of treatment, mice were sacrificed and bones, spleen and peripheral blood were harvested for further processing.

### In vivo homing assay

NSG mice were treated with daratumumab or IgG_1_ (8 mg/kg), 24 h later mice were transplanted with primary human AML cells. After 16 h, mice were sacrificed and leukemic infiltration in bone marrow, spleen and peripheral blood was assessed by flow cytometry.

### Statistical analyses

All data are shown as mean ± SEM. Shapiro–Wilk test was applied as a test of normality. In case of normality, Student´s t test was applied for comparisons between two groups. Mann–Whitney U test and Wilcoxon signed-rank test were applied for nonparametric unpaired and paired analyses. Multiple comparisons were calculated using the one-way ANOVA test. Analyses were performed with GraphPad Prism software (San Diego, USA). *p < 0.05, **p < 0.01, ***p < 0.001, ****p < 0.0001.

## Supplementary Information

Supplementary Information.

## Data Availability

The datasets generated during the current study are available from the corresponding author on reasonable request.
