# Supplementary material for: Targeting CD38 in acute myeloid leukemia interferes with leukemia trafficking and induces phagocytosis
Source: Sci Rep. 2021 Nov 11;11:22062. doi: 10.1038/s41598-021-01300-8 (PMC8586007; doi:10.1038/s41598-021-01300-8)
Supplement: Supplementary file 1 — Supplementary Information. [file 41598_2021_1300_MOESM1_ESM.pdf]

Supplementary Information

Supplementary Methods

***In vitro* experiments.** Kasumi-1, OCI-AML-3, MOLM-13, OCI M2, K562, KG-1 and THP-1 cells were purchased from the German Collection of Microorganisms and Cell Cultures (DSMZ) and cultured according to vendor´s recommendations. THP-1 cells were cultured in RPMI-1640 medium (Thermo Fisher Scientific, Waltham, MA, USA) containing 10% heat inactivated FBS (PAN Biotech, Aidenbach, Germany) and 1% penicillin/streptomycin (Sigma Aldrich, St. Louis, MOUSA). KG-1, HL-60, OCI-M2 and K562 cells were cultured in IMDM medium (Thermo Fisher Scientific) containing 20% heat inactivated FBS and 1% Penicillin/Streptomycin. Kasumi-1 and MOLM-13 cells were cultured in RPMI-1640 medium containing 20% heat inactivated FBS and 1% Penicillin/Streptomycin. OCI-AML3 cells were cultured in αMEM medium (Thermo Fisher Scientific) containing 20% heat inactivated FBS and 1% Penicillin/Streptomycin. MS-5 cells were cultured in IMDM medium supplemented with 20% FCS and 1% Penicillin/Streptomycin. HUVEC were a gift from Prof. Bernd Giebel, University of Duisburg-Essen, Essen, Germany and cultured in supplemented endothelial cell growth medium-2 (EGM2; Lonza, Basel, Switzerland and PromoCell, Heidelberg, Germany). Immortalized human bone marrow mesenchymal stem and progenitor cells (HuMSPCs) were a gift from Prof. Bernd Giebel, University of Duisburg-Essen, Essen, Germany and were previously characterized (1). HuMSPCs were cultured in IMDM medium containing 20% heat inactivated FBS, 1% Penicillin/Streptomycin and 2mM L-glutamine (Thermo Fisher Scientific). To obtain mesenpheres, HuMSCPs were cultured in DMEM/F12, human endothelial serum free medium (Thermo Fisher Scientific), 15% chicken embryo extract (US Biological, Salem, MA, USA), 0.1mM β-Mercaptoethanol (Invitrogen, Carlsbad, CA USA), 1% penicillin/streptomycin (Sigma Aldrich), 1% non-essential aminoacids (Invitrogen), 1% Nitrogen and 2% B-27 Supplement as well as 2mM fibroblast growth factor (FGF) (PeproTech, London, UK), 2mM platelet derived growth factor (PDGF) (PeproTech), 2mM oncostatin M (PeproTech), 4mM insulin like growth factor 1 (IGF-1) (PeproTech) and 2mM epidermal growth factor (EGF) (PeproTech) at clonal density. Primary hematopoietic cells were cultured in StemSpan^{TM} serum-free medium (STEMCELL Technologies, Vancouver, Canada) supplemented with recombinant human SCF (50ng/ml), TPO (50ng/ml), IL-3 (5ng/ml), IL-6 (5ng/ml) (all from PeproTech), human High Density

Lipoprotein (40µg/ml) (Millipore, Burlington, VT, USA) and Primocin (2µl/ml) (InvivoGen, San Diego, CA, USA). Daratumumab (Janssen, Beerse, Belgium), Isatuximab (Sanofi, Vitry-sur-Seine, France), IgG_1 control antibody (Sigma Aldrich) or tretinoin (all-trans retinoic acid, ATRA) (Sigma Aldrich) were added at the indicated concentrations. For all cell lines, mycoplasma contamination was excluded regularly. All cell lines were kept for maximum 5 passages in culture. To quantify hematopoietic cells after co-culture, adherent layer was trypsinized and added to non-adherent cells, live cells were quantified by Trypan Blue staining and analyzed by flow cytometry for CD45 expression. Absolute numbers of hematopoietic and stromal cells were given.

Antibody-dependent mechanisms of cytotoxicity. For evaluation of antibody dependent cell mediated cytotoxicity (ADCC), AML cells were stained with 1µM calcein-AM (Thermo Fisher Scientific) for 45 minutes at 37°C, and then washed 3 times in phosphate buffered saline (PBS) (Thermo Fisher Scientific). Target cells were incubated in daratumumab respectively IgG_1 control (0.1-10µg/ml) for 4 hours in serum-free culture medium supplemented with mononuclear cells of healthy donors as effector cells in an effector to target cell ratio of 50:1. Afterwards, supernatant was collected and Calcein-fluorescence was measured by spectrophotometry (Synergy 2, BioTek, Winooski, VT USA). Lysis was calculated by setting a range of 0 to 100% using conditioned medium as minimal and media supplemented with Triton X-100 as maximal control. Complement dependent cytotoxicity (CDC) was measured by staining AML cells with calcein-AM as described above. Stained cells were incubated in medium supplemented with 10% normal human serum (Sigma-Aldrich) with daratumumab or IgG_1. One hour later, fluorescence of supernatant was measured by spectrophotometry. Lysis was calculated by setting a range of 0 to 100% using conditioned medium as minimal and media supplemented with Triton X-100 as maximal control. For antibody-dependent phagocytosis (ADCP) assays, primary human macrophages were obtained by magnetic cell isolation (Miltenyi, Bergisch Gladbach, Germany) of $CD14^+$ monocytes from peripheral blood mononuclear cells of healthy donors. Monocytes were differentiated to adherent macrophages over 6 days using 100ng/ml macrophage colony-stimulating factor (M-CSF, Peprotech, London, UK). Macrophages were then seeded and allowed to adhere overnight. Calcein-AM stained AML cells (target cells) were added to macrophages in a 2:1 effector to target cell ratio. Daratumumab or IgG_1 (0-10µg/ml) was added for 4 hours. Phagocytosed

AML cells were detected by co-expression of CD16 and calcein-AM expressing AML cells by flow cytometry.

Mitochondrial Trafficking. To test transfer of mitochondria between cell fractions in co-culture experiments, either stroma or AML cells were stained with 200nM MitoTracker™ Green FM (Thermo Fisher Scientific) for 60 minutes, washed 3 times with PBS and co-cultured in presence of either daratumumab or IgG_1 control (0,1µg/ml) for 24 hours. Mitochondria transfer was tracked by flow cytometry. For mtDNA quantification, AML cells were sorted after co-culture and expression of either human or mouse mitochondrial DNA was analyzed by RT-PCR. Amount of mitochondrial DNA was related to nuclear encoded B2M.

Migration assay. To assess transendothelial migration capacity of primary AML cells, HUVEC were seeded into 24mm transwell inserts (Corning© Transwell© polycarbonate 8µm pore, Corning, NY, USA) at a concentration of $5x10^5$ per well. After reaching confluency, HUVEC were pretreated with 20ng/ml human TNF-alpha and incubated overnight. $1x10^6$ primary AML cells were added into transwell inserts and treated with either 0,1µg/ml daratumumab or IgG_1 control. Lower chamber was supplemented with cell culture medium. After 16 hours, medium of lower chamber was removed and migrated cells were counted and characterized by flow cytometry. Calculation of migration index: cells migrated to the lower chamber/total cells seeded.

Histopathologic Analysis. Femur and spleen samples were fixed in 4% paraformaldehyde overnight, bones were decalcified for 3-5 days using OSTEOSOFT® (Merck, Kenilworth, NJ, USA). Afterwards, tissues were dehydrated in increasing concentrations of ethanol and embedded in paraffin. Paraffin sections were cut on a rotary microtome and stained either automatically for hematoxylin and eosin (H&E) or manually for reticulum staining with Gomori´s silver impregnation method. Slides were imaged with a Zeiss AxioObserver Z1 microscope with Apotome (Zeiss, Oberkochen, Germany).

Flow Cytometry. Fluorochrome-conjugated monoclonal antibodies specific to mouse CD45 (30-F11) or human CD45 (HI30), CD38 (HB7), CD33 (WM-53) from eBioscience (Thermo Fisher Scientific), monoclonal antibodies specific to human CD16 (3G8), CD3 (UCHT1), CD144 (55-7H1) from BD Pharmingen (BD Biosciences, Franklin Lakes, NJ USA) were used. Positive specific antibody labeling was gated in reference to

corresponding isotype control or fluorescence minus one (FMO) corresponding sample. Multiparameter analyses of stained cell suspensions were performed on a BD LSRII flow cytometer (BD Biosciences) and analyzed with FlowJo software (FlowJo LLC, Ashland, OR, USA); cell sorting was performed with a BD FACSAria III (BD Biosciences). DAPI^{-} single cells were evaluated for all analyses. For flow cytometric analysis of apoptosis, cells were stained with Annexin V and DAPI using the Annexin V Apoptosis Detection Kit I (BD Biosciences). Proliferation assays were performed using the BrdU Flow Kit (BD Biosciences) according to manufacturer's instructions.

RT-PCR. Freshly sorted cells were collected in lysis buffer and RNA was isolated using Dynabeads® mRNA DIRECT™ Purification Kit (Invitrogen, Carlsbad, CA, USA) according to manufacturer's instructions. Reverse transcription was performed using RNA to cDNA EcoDry™ Premix 3 system (Clontech, Mountain View, CA, USA). Quantitative real-time PCR was performed on an ABI PRISM 7900HT Sequence Detection System (Applied Biosystems, Foster City, CA USA). The relative mRNA abundance was calculated using the ΔCT method. Gene expression data was normalized to *GAPDH.* Primer sequences are included below.

**Gene symbol**	**Sequence forward**	**Sequence reverse**
GAPDH	TCTGCTCCTCCTGTTCGACA	AAAAGCAGCCCTGGTGACC
ANGPT1	GCCATCTCCGACTTCATGTT	CTGCAGAGAGATGCTCCACA
APAF1	AAGGTGGAGTACCACAGAGG	TCCATGTATGGTGACCCATCC
BAX	CCCGAGAGGTCTTTTTCCGAG	CCAGCCCATGATGGTTCTGAT
BCL2	GGTGGGGTCATGTGTGTGG	CGGTTCAGGTACTCAGTCATCC
CASP3	CATGGAAGCGAATCAATGGACT	CTGTACCAGACCGAGATGTCA
CASP8	TTTCTGCCTACAGGGTCATGC	GCTGCTTCTCTCTTTGCTGAA
CASP9	CTTCGTTTCTGCGAACTAACAGG	GCACCACTGGGGTAAGGTTT
CDH5	TTGGAACCAGATGCACATTGAT	TCTTGCGACTCACGCTTGAC
CD38	CAACTCTGTCTTGGCGTCAGT	CCCATACACTTTGGCAGTCTACA
CXCL12	TGGGCTCCTACTGTAAGGGTT	TTGACCCGAAGCTAAAGTGG
CXCR4	ACTACACCGAGGAAATGGGCT	CCCACAATGCCAGTTAAGAAGA
CYTC	CTTTGGGCGGAAGACAGGTC	TTATTGGCGGCTGTGTAAGAG
ICAM1	ATGCCCAGACATCTGTGTCC	GGGGTCTCTATGCCCAACAA

MCL1	TGCTTCGGAAACTGGACATCA	TAGCCACAAAGGCACCAAAAG
PECAM1	AACAGTGTTGACATGAAGAGCC	TGTAAAACAGCACGTCATCCTT
SELE	AGAGTGGAGCCTGGTCTTACA	CCTTTGCTGACAATAAGCACTGG
BID	ATGGACCGTAGCATCCCTCC	GTAGGTGCGTAGGTTCTGGT
VCAM1	GTCTCCAATCTGAGCAGCAA	TGAGGATGGAAGATTCTGGA
VEGFA	AGGGCAGAATCATCACGAAGT	AGGGTCTCGATTGGATGGCA
nuclear-B2M	TGCTGTCTCCATGTTTGATGTATCT	TCTCTGCTCCCCACCTCTAAGT
human mitochondrial tRNA-LEU	CACCCAAGAACAGGGTTTGT	TGGCCATGGGTATGTTGTTA
mouse mitochondrial Cytochrome B	CTTCATGTCGGACGAGGCTTA	TGTGGCTATGACTGCGAACA

Supplementary Figures

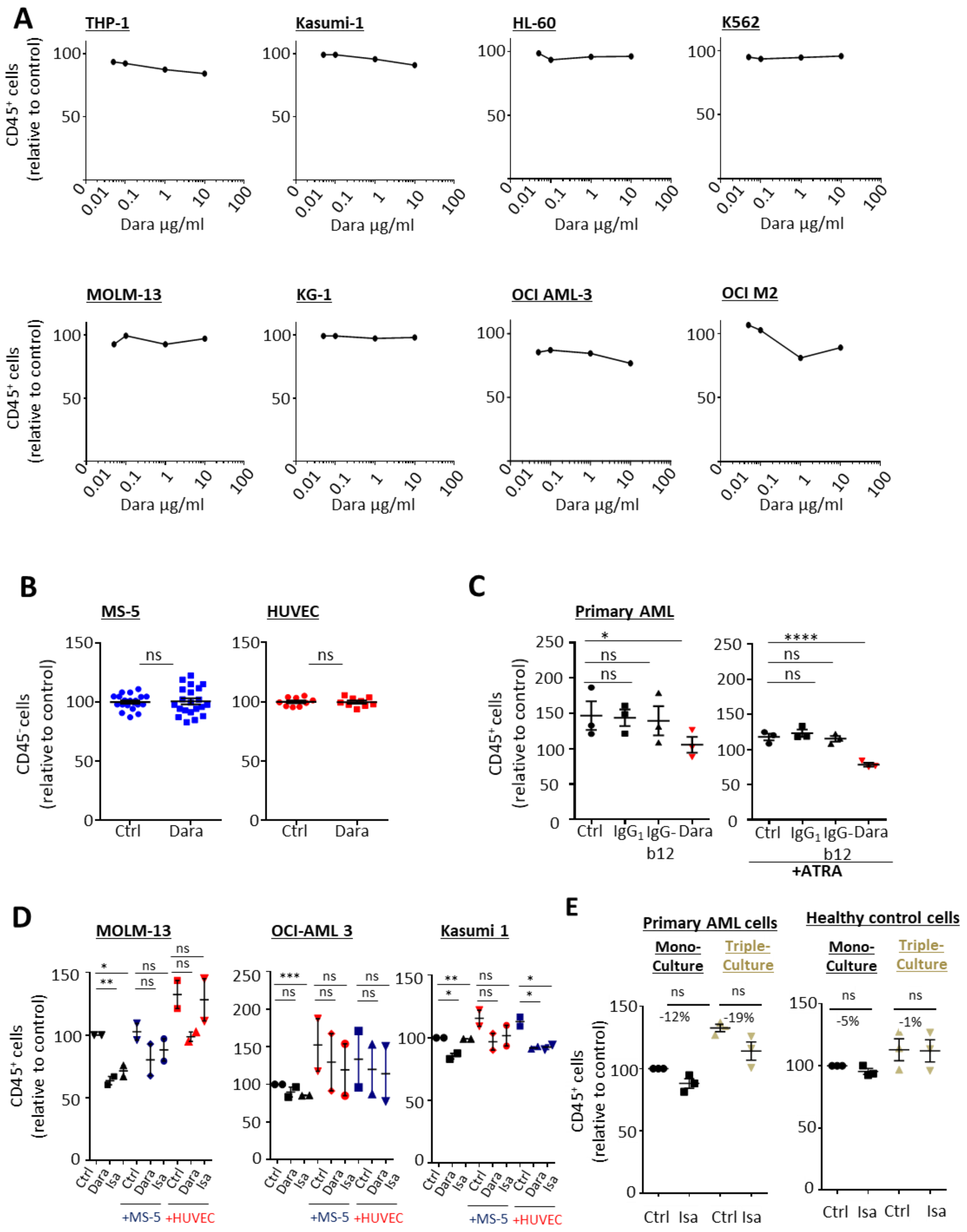

Supplementary Figure S1. CD38 inhibition results in high anti-leukemic efficacy *in vitro* independent of CD38 expression level. A, mean cell reduction after

treatment of different human AML cell lines with increasing concentration of daratumumab (0 µg/ml, 0.01µg/ml, 0.1µg/ml, 1µg/ml, 10µg/ml) (n=3). **B,** quantification of MS-5 (n=7 independent experiments) and HUVEC cells (n=3 independent experiments) after treatment with either 0.1µg/ml daratumumab or vehicle for 4 days. Absolute numbers of $CD45^-$ cells were normalized to control. **C,** Primary AML cells (n=3) were mono-, co- or triple-cultured with HUVEC and/or mesenspheres and treated with 0.1µg/ml daratumumab, vehicle or both antibody controls (IgG-b12 and IgG_1) for 3 days. Absolute numbers of $CD45^+$ cells were normalized to mono-cultured control. Each dot represents the mean of triplicates. **D**, human AML cell lines were either mono- or co-cultured with MS-5 stroma cells or HUVEC cells and treated with 0.1µg/ml daratumumab, 10µg/ml isatuximab or IgG_1 control for 3 days. Absolute numbers of $CD45^+$ cells were normalized to mono-cultured control. Each dot represents the mean of triplicates. **E**, primary AML cells (n=3) or healthy peripheral blood mononuclear cells (n=3) were mono- or triple-cultured with HUVEC and mesenspheres and treated with either 10µg/ml isatuximab or IgG_1 control for 3 days. Absolute numbers of $CD45^+$ cells were normalized to mono-cultured control. Each dot represents the mean of triplicates, for each pair the mean change in cell count is given in percent. Data are shown as mean ± SEM. n.s., not significant, *$p<0.05$, **$p<0.01$ ***$p<0.001$ ****$p<0.0001$ as determined by unpaired t-test (B,D), paired one way ANOVA (C) and Wilcoxon signed-rank test (E).

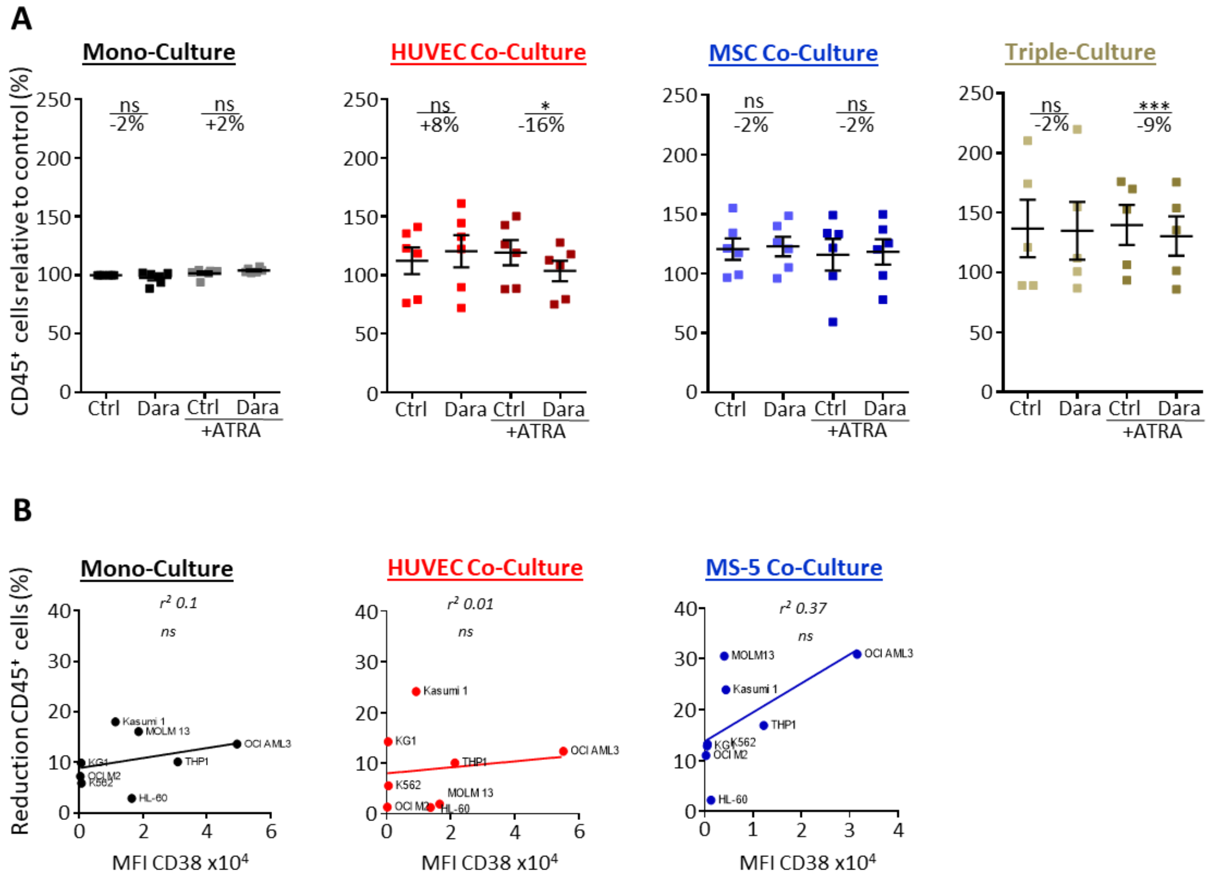

Supplementary Figure S2. CD38 expression of AML cells is regulated by microenvironmental cues and induced upon ATRA treatment. A, healthy peripheral blood mononuclear cells (n=6 different donors) were mono-, co- or triple-cultured with HUVEC and/or mesenspheres. All-trans-retinoic acid (ATRA) or vehicle was added at 0.1µM for 2 days followed by daratumumab treatment at 0.1µg/ml or vehicle for another 3 days. Absolute numbers of CD45$^+$ cells were normalized to mono-cultured control. Each dot represents the mean of triplicates. **B,** correlation of anti-leukemic activity of daratumumab given as mean cell-count reduction with CD38 expression given as mean fluorescence intensity (MFI) measured by flow cytometry for the indicated AML cell line from co-culture experiments with MS-5 or HUVEC (n= 3 independent experiments). Data are shown as mean ± SEM. n.s., not significant, *p<0.05, ***p<0.001 as determined by Wilcoxon signed-rank test (A) and linear regression (B).

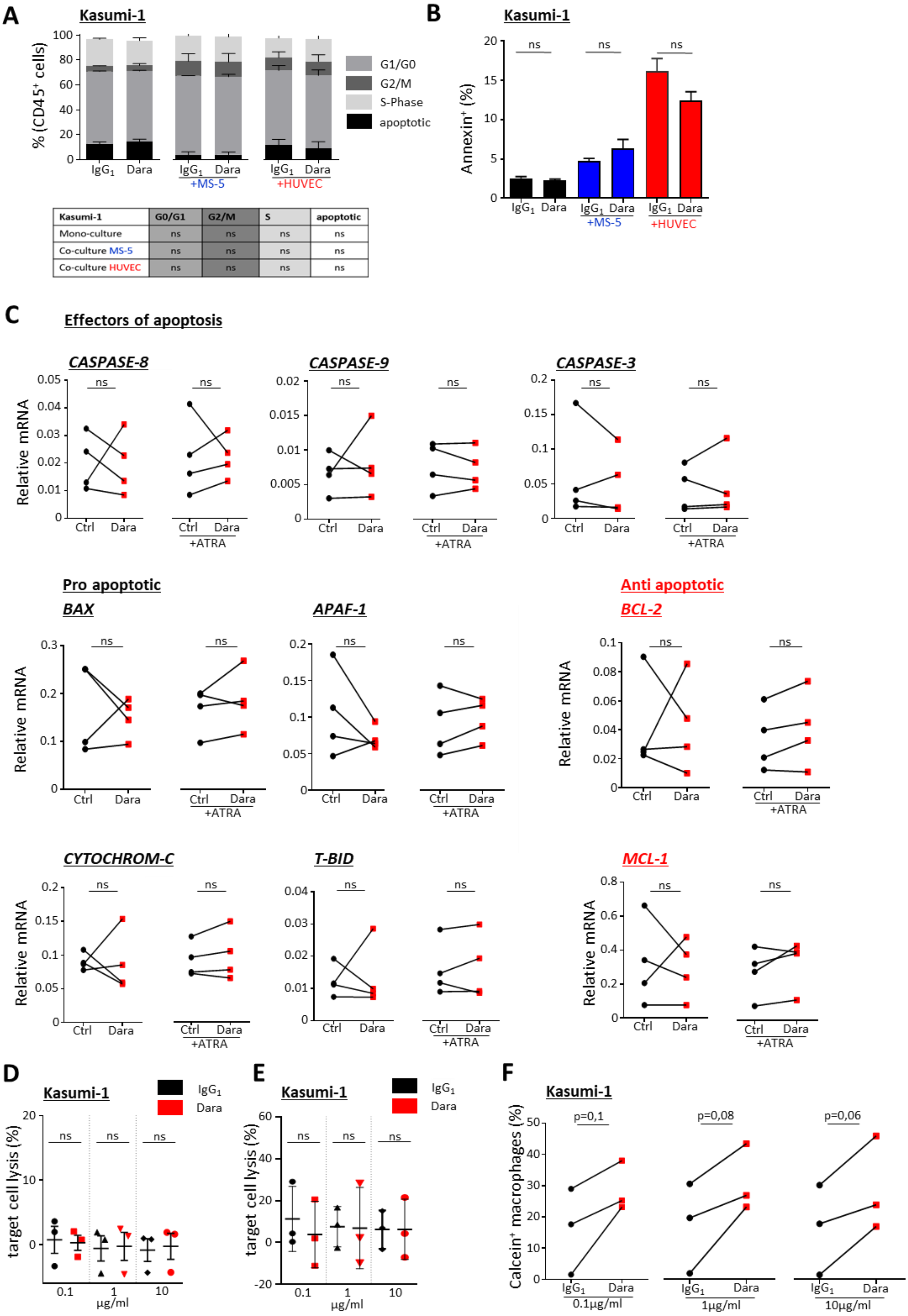

Kasumi-1	G0/G1	G2/M	S	apoptotic
Mono-culture	ns	ns	ns	ns
Co-culture MS-5	ns	ns	ns	ns
Co-culture HUVEC	ns	ns	ns	ns

Supplementary Figure S3. Daratumumab induces phagocytosis in AML. A, cell proliferation analysis using BD bromodeoxyuridine (BrdU) flow assay. Quantification for Kasumi-1 cells mono- or co-cultured with MS-5 or HUVEC after treatment with 0.1µg/ml daratumumab or IgG_1-control for 4 days, statistical analysis is given below (n= 3 independent experiments). **B,** quantification of apoptotic cells by Annexin V assay in Kasumi-1 cells mono- or co-cultured with MS-5 or HUVEC after treatment with 0.1µg/ml daratumumab or IgG_1-control for 4 days (n= 3 independent experiments). **C**, triple-cultured primary AML cells (n=5) were sorted and mRNA expression levels of different key genes related to apoptosis were measured by rt-PCR. Shown are relative mRNA related to housekeeping gene *GAPDH*. Each dot represents the mean of triplicates. **D,** quantification of complement-dependent-cytotoxicity (CDC). Calcein-AM stained Kasumi-1 cells were incubated in medium supplemented with 10% normal human serum and treated with increasing dosages of daratumumab or IgG_1-control for 1 hour. Calcein-fluorescence was measured in supernatant by spectrophotometry. Lysis was calculated by using detergent as maximum and medium as minimum control (n= 3 independent experiments). **E,** quantification of antibody-dependent cell-mediated cytotoxicity (ADCC) calcein-AM stained Kasumi-1 cells were incubated with effector cells and treated with increasing dosages of daratumumab or IgG_1-control. Calcein-fluorescence was measured in supernatant after 4 hours by spectrophotometry. Lysis was calculated by using detergent as maximum and medium as minimum control (n= 3 independent experiments). **F,** quantification of antibody-dependent phagocytosis (ADCP). Macrophages were cultured with calcein-AM stained Kasumi-1 cells (n=3 independent experiments) and treated with increasing dosages of daratumumab or IgG_1 control. $CD16^+$ $calcein^+$ phagocyting macrophages were measured by flow-cytometry. Each dot represents the mean of triplicates. Data are shown as mean ± SEM. n.s., not significant as determined by unpaired student´s t-test (A, B, D, E, F) and Wilcoxon signed-rank test (C).

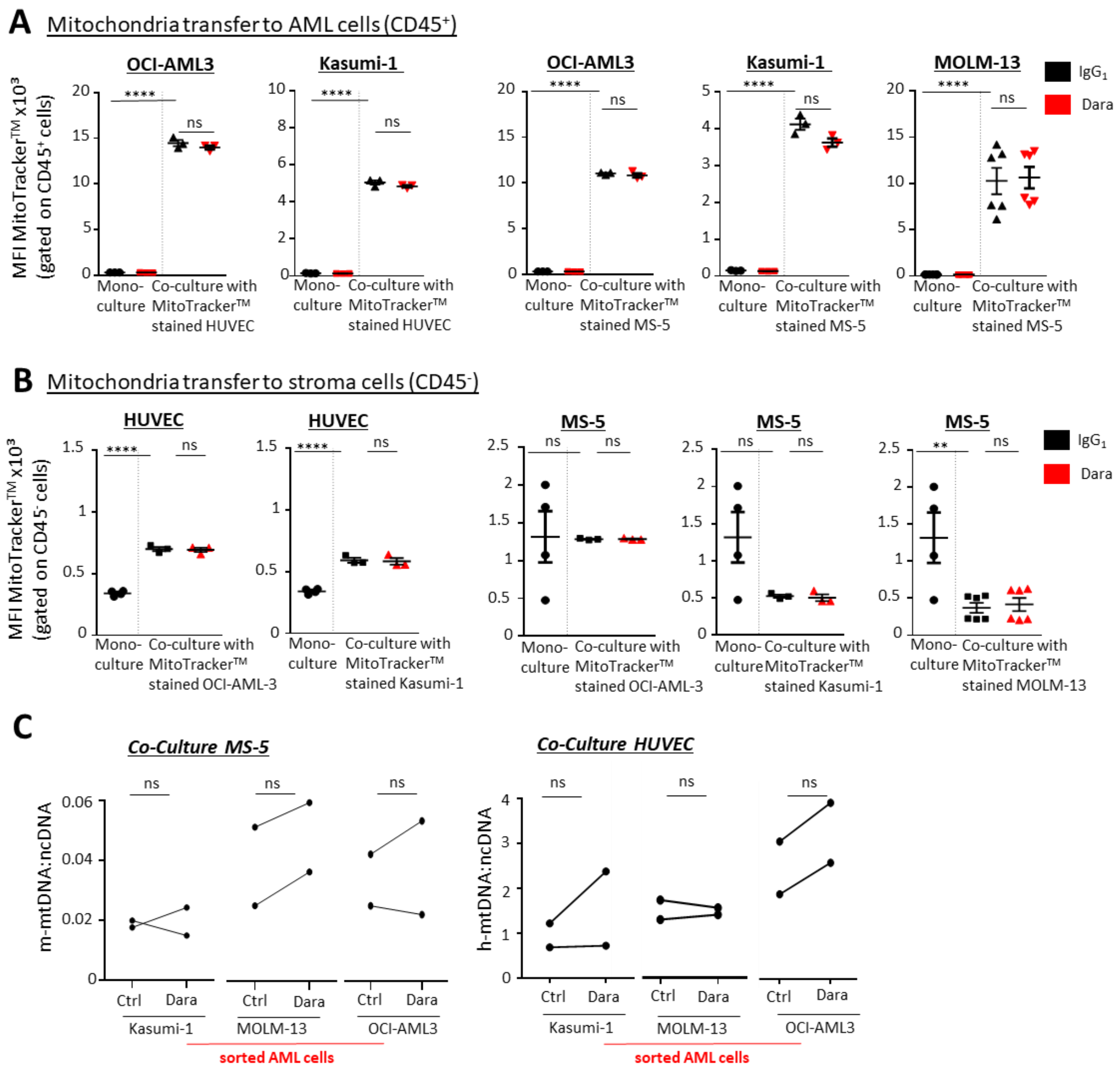

Supplementary Figure S4. Daratumumab does not interfere in mitochondria transfer in the microenvironment. A-B, tracking mitochondrial transfer in co-culture was performed by staining mitochondria with MitoTracker™. **A,** mean fluorescence intensity (MFI) of MitoTracker™ in CD45+ AML cells after co-culture with MitoTracker™ stained HUVEC or MS-5 cells with unstained AML cells and treatment with 0.1µg/ml daratumumab or IgG1 control for 2 days or mono-culture of unstained AML cells as control. **B,** MFI of MitoTracker™ in CD45- stroma cells after co-culture with MitoTracker™ stained AML cells with indicated stroma cells and treating with 0.1µg/ml daratumumab or IgG_1 control for 2 days or mono-culture of unstained stroma cells as control. **C,** Indicated AML cells were co-cultured with either HUVEC or MS-5 cells for 3 days, sorted by flow cytometry and mRNA expression levels were measured by rt-PCR for either human mitochondrial DNA (hmtDNA) after HUVEC co-culture or mouse

mitochondrial DNA (mmtDNA) after MS-5 co-culture, relative to ncDNA. Data are shown as mean ± SEM. n.s., not significant, $^{**}p<0.01$, $^{***}p<0.0001$ as determined by unpaired student´s t-test (A,B) or Mann-Whitney-U-test (C).

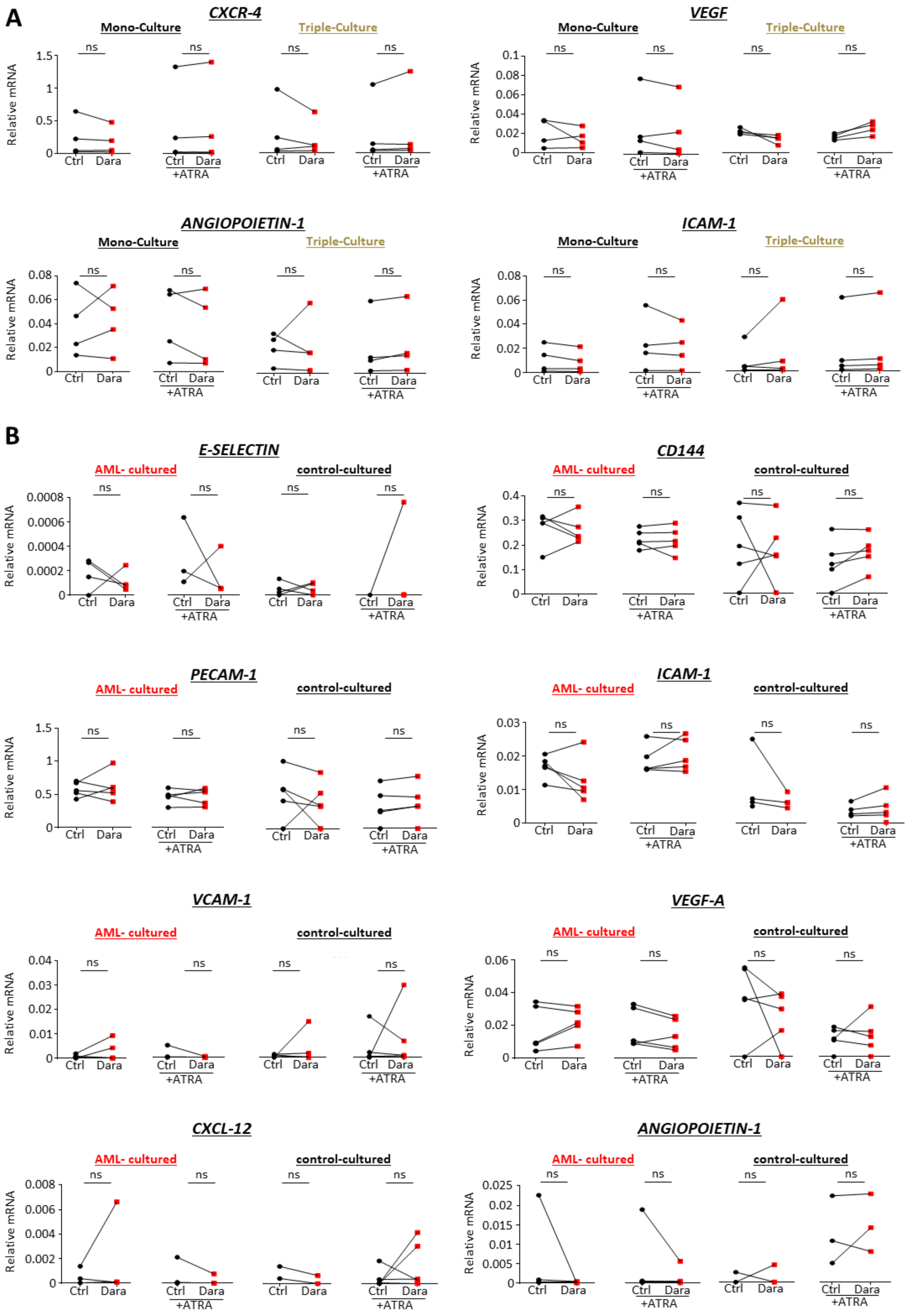

A
CXCR-4
Mono-Culture
Triple-Culture
VEGF
ANGIOPOIETIN-1
ICAM-1
Relative mRNA
ns
Ctrl
Dara
+ATRA
B
E-SELECTIN
AML- cultured
control-cultured
CD144
PECAM-1
ICAM-1
VCAM-1
VEGF-A
CXCL-12
ANGIOPOIETIN-1

Supplementary Figure S5. Daratumumab does not change the expression of genes associated with migration, retention, adhesion or angiogenesis-related factors. Triple-cultured primary AML cells (A) or $CD144^+$ endothelial cells (B) were sorted by flow cytometry and mRNA expression levels were measured by rt-PCR for migration and retention- (*CXCR4, CXCL12*), adhesion- (*ICAM-1, VCAM-1, E-selectin, PECAM-1 (CD31)*) and angiogenesis-related factors (*Angiopoietin-1, VEGF, VE-cadherin*). Shown is relative mRNA related to housekeeping gene GAPDH. Data are shown as mean ± SEM. n.s., not significant, as determined by Wilcoxon signed-rank test.

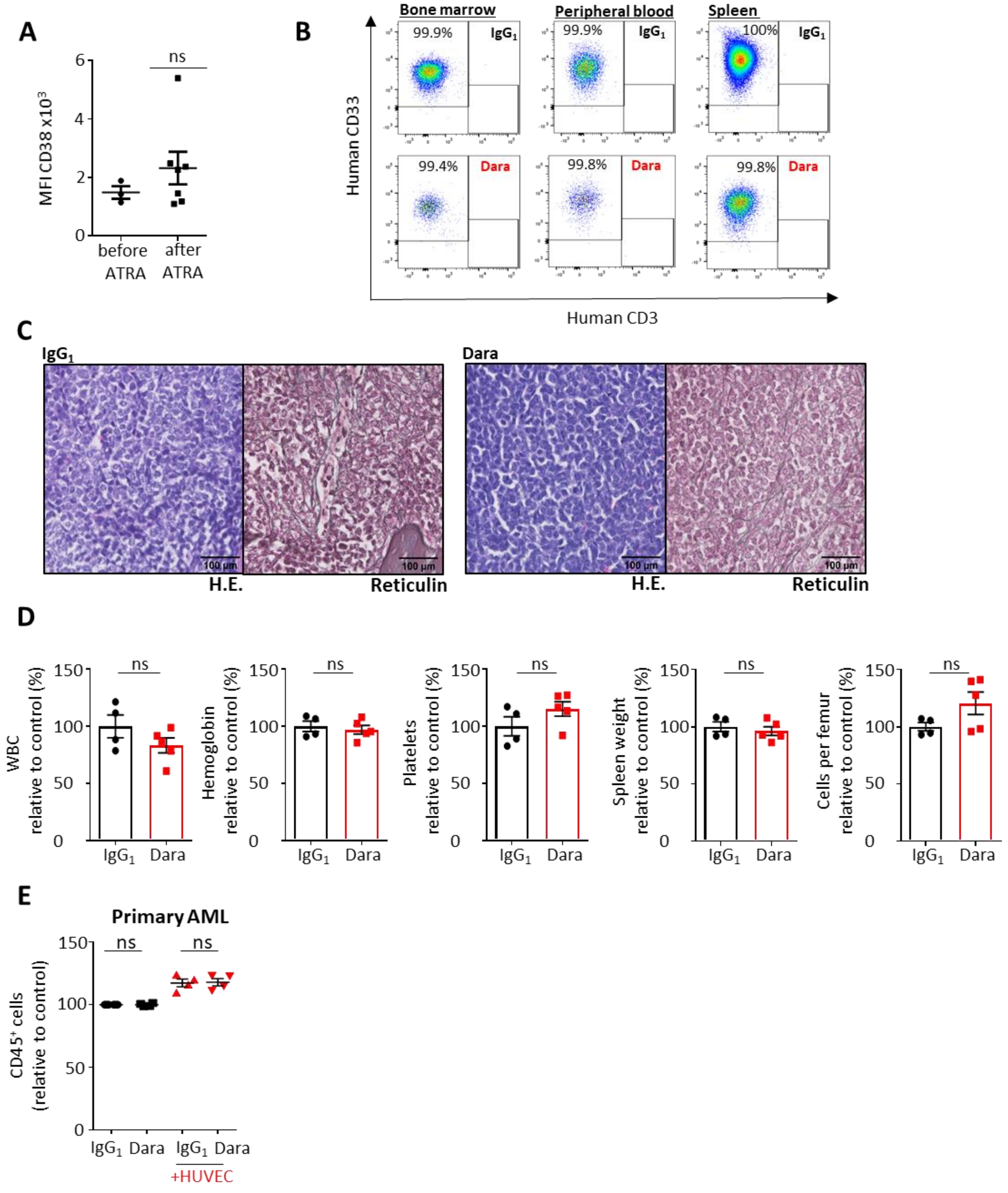

Supplementary Figure S6. Daratumumab interferes in AML cell trafficking. A, mean fluorescence intensity measured by flow cytometry of one primary AML sample before transplantation into NSG mice and after long-term treatment with ATRA harvested from murine bone marrow. **B**, Representative flow cytometry plots of CD45$^+$ AML cells (from Figure 4B) showing exclusive engraftment of myeloid blasts. **C,** representative images of Hematoxylin and Eosin (HE) and reticulum staining on femur

sections after long-term treatment with daratumumab or IgG_1 and ATRA. Magnification 40x, scale bar 100µm. **D,** peripheral blood counts, hemoglobin, wet spleen weight and cells per femur of cytarabine, daratumumab / IgG_1 treated mice (data normalized to control, n=9. **E**, primary AML cells were mono- or co-cultured with HUVEC, treated with 0.1µg/ml daratumumab or IgG1 control for 16 hours. Absolute numbers of $CD45^+$ cells were normalized to mono-cultured control. Each dot represents the mean of triplicates. Data are shown as mean ± SEM. n.s., not significant as determined by unpaired t-test (A, E) and Wilcoxon signed-rank test (D).

Supplementary Table

sample	ELN-genetic risk stratification	cytogenetic characteristics	molecular characteristics	CD33 expression [%]	CD38 expression [%]
1	adverse	complex aberrant karyotype		97,1	21,9
2	adverse	45,XX, t(3,3)(q21;q26),-7	rearrangement in EVI1-gene region	97,7	98,1
3	adverse	46,XX	FLT3-LM	99,4	100
4	intermediate	46,XY	no mutations	82	83
5	favorable	46,XX	NPM-1	99,5	99.7
6	adverse	45,XX, -7	no mutations	77	85
7	favorable	46, XX	KMT2A-PTD, NPM-1, FLT3-ITD (Ratio 0,39)	95	99,3
8	n.a.	46,XX[25]	n.a.	96	96
9	favorable	46,XY	IDH2, NPM1, FLT3-ITD (Ratio 0,09)	99	96
10	favorable	46,XY	NPM-1		98,3
11	adverse	complex aberrant karyotype	TP-53 (missense mutation c.659A>G in p.Tyr220Cys)	94,3	97,3
12	adverse	46,XX	FLT3-ITD (0,7)	97.8	99,8
13	intermediate	46, XX	FLT-3 (Ratio 0,57), NPM-1	100	80
14	favorable	46,XY [12]	NPM 1, FLT3-TKD	93,6	96,2
15	adverse	46, XY, -7,+19 [25]	FLT3-TKD	100	0
16	intermediate	46,XX	FLT3-LM (ratio 0,391), NPM-1	99	97,4
17	favorable	46,XX	NPM-1	98	95

Supplemental Table 1. Patient characteristics. ELN genetic risk stratification, cytogenetic and molecular characteristics as well as CD33 and CD38 expression of blasts of primary human AML samples.

References

1. Chen Y, Hoffmeister LM, Zaun Y, Arnold L, Schmid KW, Giebel B, Klein-Hitpass L, Hanenberg H, Squire A, Reinhardt HC, Duhrsen U, Bertram S, Hanoun M. Acute myeloid leukemia-induced remodeling of the human bone marrow niche predicts clinical outcome. Blood Adv. 2020 Oct 27;4(20):5257-5268. Epub 2020/10/28. doi:10.1182/bloodadvances.2020001808. Cited in: Pubmed; PMID 33108453.
